# Potassium and Cesium Fluorinated β-Diketonates: Effect of a Cation and Terminal Substituent on Structural and Thermal Properties

**DOI:** 10.3390/molecules28155886

**Published:** 2023-08-04

**Authors:** Danil V. Kochelakov, Evgeniia S. Vikulova, Natalia V. Kuratieva, Ilya V. Korolkov

**Affiliations:** Nikolaev Institute of Inorganic Chemistry, Siberian Branch of Russian Academy of Sciences, Acadademic Lavrentiev Ave 3, 630090 Novosibirsk, Russia; kuratieva@gmail.com

**Keywords:** alkali metals, beta-diketonates, fluorinated complexes, polyhedra, thermal analysis

## Abstract

As potential precursors for the synthesis of fluoroperovskites, a family of heavy alkali metal (M^I^ = K, Cs) fluorinated β-diketonates were prepared and characterized by elemental analysis, IR, and powder-XRD. The crystal structures of the new six complexes, M^I^(β-dik^F^)(H_2_O)_X_, X = 0 or 1, were also determined. The structural diversity of this poorly explored class of complexes was discussed, including the preferred types of cation polyhedra and the ligand coordination modes, and the thermal properties of the metal β-diketonates were studied by TG–DTA in an inert (He) atmosphere. The data obtained allowed us to reveal the effect of the metal cation and the terminal substituent on the structural and thermal features of this family of complexes.

## 1. Introduction

The steady interest in the metal fluorinated β-diketonates is associated with a variety of their properties which are dependent on the structure of the carbon skeleton of the anion (R^1^C(O)CHC(O)CR^2^)^−^ = (R^1^..R^2^)^−^ in which there are two sites for varying substituents. As a consequence, compounds in this class are used in a large range of applications, such as catalysis [1], optoelectronic devices [2], and volatile precursors for the synthesis of functional materials [3].

However, alkali metal β-diketonates M^I^(R^1^..R^2^) are still a “blank spot on the map” of this area of coordination chemistry. This is related to the fact that they have been traditionally considered mainly as sources of ligands in the synthesis of complexes of other metals [4,5,6,7]. Nowadays, β-diketonates M^I^(R^1^..R^2^) have attracted attention as the precursors for fluoroperovskite M^I^M^II^F_3_ (M^I^ = alkali metal; M^II^ = bivalent metal), which has drawn greater interest owing to their physical and chemical characteristics [4,8,9]. For example, *M*CaF_3_ (*M* = K, Rb, Cs) has been the subject of study for transparent optical coatings [10] and phosphors for radiation dosimetry when doped with lanthanide ions [11]. *M*MgF_3_ plays an important role as a scintillator [12], luminescent host material [13,14], and laser [15]. *M*MnF_3_ (*M* = K, Rb, Cs) serves as a light upconversion host [16] and antiferromagnet [17,18]. However, the preparation of fluoroperovskites by the decomposition of metal–organic precursors such as homo and heterometallic β-diketonates requires milder conditions [19] than conventional direct synthesis from metal fluorides [20,21,22].

In addition, potassium and cesium β-diketonates are being actively developed as precursors for other functional materials. For example, cesium hexafluoroacetylacetonate, Cs(CF_3_..CF_3_), was successfully applied for the green solution synthesis of perovskites CsPbBr_3_ and CsPb_2_Br_5_ [23], with Br_2_ as the precipitating agent. Additionally, heteroligand complexes based on potassium hexafluoroacetylacetonate, e.g., K(CF_3_..CF_3_)(glyme) (glyme = 2,5,8,11,14-pentaoxapentadecane), were recently developed for solution-based methods of fabrication of KF thin films [24].

The features of heavy alkali metals (HAM, *M* = K, Rb, Cs) include high and inconstant coordination numbers and a low charge density. These peculiarities, combined with the variety of coordination modes of fluorinated β-diketonate ions, including the chelating or bridging functions of oxygen/fluorine atoms, lead to the structural flexibility of the corresponding complexes. In turn, the structure largely determines the utility characteristics such as solubility, thermal properties, and others. Therefore, varying the substituents (R^1^ and R^2^) in the β-dicarbonyl fragment is an effective tool for fine-tuning the physicochemical properties of this class of precursors. However, this requires the revealing of the fundamental relationships between the structure of complexes and their functional characteristics, i.e., the supplementation and systematization of relevant data. Previous studies were focused mostly on the effects of a non-fluorinated substituent with a fixed CF_3_ group [25,26,27] or non-fluorinated complexes [28]. In this work, we continue the systematic study of alkali metal β-diketonates and analyze the effect of an increase in the length of the fluorinated substituent (from C_2_F_5_ to C_3_F_7_).

**Figure 1:**
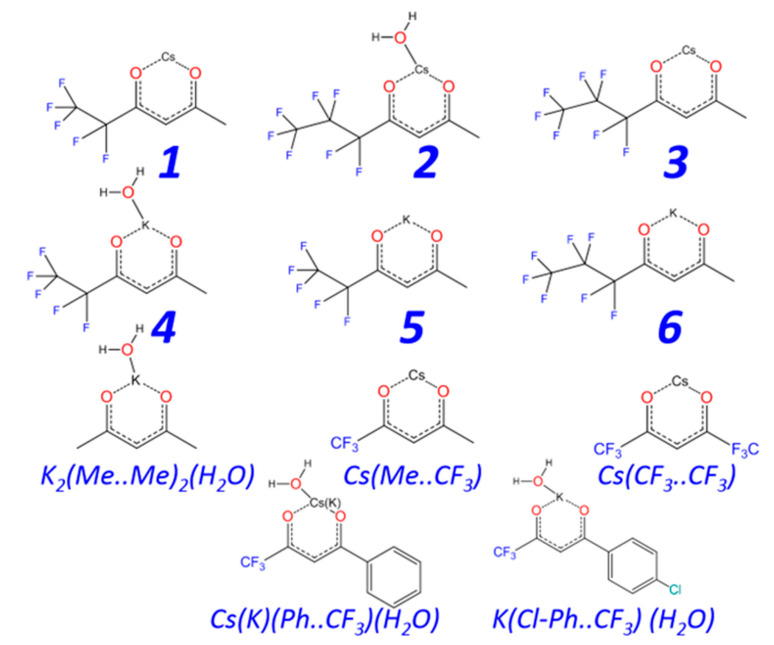
The stoichiometric schemes synthesized in this work (numbered designations) and previously known (letter designations) and cesium and potassium β-diketonate complexes.

Here, we report on the synthesis of novel potassium and cesium complexes of the type “*M*(Me..C_2_F_5_)(H_2_O)_X_” or “*M*(Me..C_3_F_7_)(H_2_O)_X_”, (*M* = K, Cs; (Me..C_2_F_5_) = 1,1,1,2,2-pentafluoro-3,5-hexanedionate; (Me..C_3_F_7_) = 1,1,1,2,2,3,3-heptafluoro-4,6-heptanedionate, X = 0, 1, Figure 1). The compounds are then characterized by FT-IR spectroscopy, elemental analysis, TG–DTA, and X-ray single-crystal (SXRD) and powder (PXRD) diffraction.

## 2. Results and Discussion

### 2.1. Synthesis and Characterization

All individual β-diketonates M^I^(Me..C_2_F_5_)(H_2_O)_X_ and M^I^(Me..C_3_F_7_)(H_2_O) (M^I^ = K, Cs) **1**–**6** were obtained for the first time. Due to the high acidity of fluorinated β-diketones [29], it was possible to use a simple synthetic method through the neutralization of a metal carbonate at room temperature [25,26,27]. However, water is additionally formed as a product, which can potentially be included in the structure of the complexes. The latter is expected to be dependent both on the β-diketonate ligand and HAM cation, but the available data are not yet sufficient to identify general trends. On the one hand, the works [25,27] demonstrated the absence of water in the structure of metal hexafluoroacetylacetonates (*M*(CF_3_..CF_3_), *M* = K, Rb, Cs) since *M*–F contacts effectively saturated the coordination environment of the cation. On the other hand, both the non-fluorinated potassium acetylacetonate [28] and the fluorinated HAM β-diketonates with an aromatic substituent [26,30] contained water molecules in the structures. In the case of fluorinated β-diketonates with alkyl substituents, such data are fragmentary. For cesium, it was shown that such complexes could be both hydrated (R^1^ = CF_3_, R^2^ = ^t^Bu) and anhydrous (R^1^ = CF_3_, R^2^ = Me) [25].

In the current work, we demonstrated that the HAM β-diketonates with a simple alkyl (R^1^ = Me) and increased fluorinated substituents (R^1^ = C_2_F_5_, C_3_F_7_), as a rule, are characterized by their lability of water inclusion. In particular, only Cs(Me..C_2_F_5_) **1** was obtained in an anhydrous form that is stable during storage. On the contrary, the other obtained complexes can change their composition in time. The stoichiometry of Cs(Me..C_3_F_7_)(H_2_O) **2**, confirmed by powder XRD and TGA data, changes to Cs(Me..C_3_F_7_)(H_2_O)_2_ during storage, as indicated by the elemental analysis data obtained after two weeks. The diffraction pattern of **2** also changed during storage in the air (Figure S1). For K(Me..C_2_F_5_)(H_2_O) **4** and K(Me..C_3_F_7_) **6**, the elemental analysis data indicated the formation of a mixture of hydrated and anhydrous products.

These observations supported by TGA data (par. 2.3) allowed us to suggest the possibility of obtaining anhydrous complexes by room-temperature dehydration. In fact, the holding of the hydrated compounds **2** and **4** in desiccators over P_2_O_5_ for 1–2 weeks led to the formation of the anhydrous derivatives **3** and **5**, respectively. Their compositions were confirmed by IR, single-crystal, and powder XRD.

For **5**, the powder diffraction pattern also coincided with the theoretical one for the anhydrous complex and was stable during exposure to air (Figure S1). For **3**, the PXRD data showed that water molecules were rapidly included in reverse. In fact, the diffraction pattern of the as-prepared powder sample corresponded to a mixture of **2** and **3** (Figure S1). Within an hour, the sample was completely converted to hydrated complex **2** (Figure S1). Nevertheless, IR spectroscopy made it possible to fix the formation of a powder of the anhydrous complex **3**, probably due to the shorter time for recording data than for PXRD.

At the same time, no hydrated crystals were found during the selection of samples **3** and **5** for SXRD. Note that a common unpurified Et_2_O solvent was used to grow single crystals of these anhydrous compounds. This indicated the delayed character of the “reverse” inclusion of water in both single-crystal samples, probably due to their smaller surface areas. Thus, such a simple approach can be applied to the preparation of anhydrous alkali metal β-diketonates with fluorinated terminal substituents.

The presence/absence of water in the compounds was confirmed by IR spectroscopy through the presence/absence of absorption bands in the region of 3400–3100 cm^−1^ (ν(O-H)). Note that bending vibration bands δ(H-O-H) (1700–1600 cm^−1^) could not be identified due to the intersection with the region of strong absorption bands corresponding to ν(C = O) + ν(C = C) of the β-diketonate cycle (1650–1500 cm^−1^). In all the IR-spectra of samples in KBr, there were intense absorption bands in the region of 1300–1000 cm^−1^, corresponding to ν(C-F), and in the region of 3050–2900 cm^−1^, corresponding to (v(C-H)).

The comparison of the calculated and experimental XRD patterns unambiguously identified the phase composition for nearly all complexes (Figure S1). The exception observed for Cs(Me..C_3_F_7_) **3** could be associated with the composition change discussed above. Complexes **1**–**6** were soluble in polar organic solvents such as ethanol, ethyl acetate, and acetone, and were also soluble in water.

### 2.2. Crystal Structure Investigation

All the structures of **1**–**6** were determined by single-crystal XRD. The Supplementary Materials (Table S1) contain the crystallographic data and the diffraction experiment and refinement details.

To describe the cation polyhedral environment, we adopted the following criteria: K-F/K-O up to 3.10 Å and Cs-F/Cs-O up to 3.40 Å [31]. Since heavy alkali metals could form a number of different coordination polyhedra, we applied the program “SHAPE” [32] which is actively used to determine the polyhedral type of cations exhibiting large coordination numbers, e.g., lanthanides [33,34]. The results are presented in Table 1 while the continuous shapes measures (CShM) for the sets of the standard polyhedra are summarized in Table S2. In the figures below, the polyhedra with the same coordination environment are represented by the same color.

**Figure 2:**
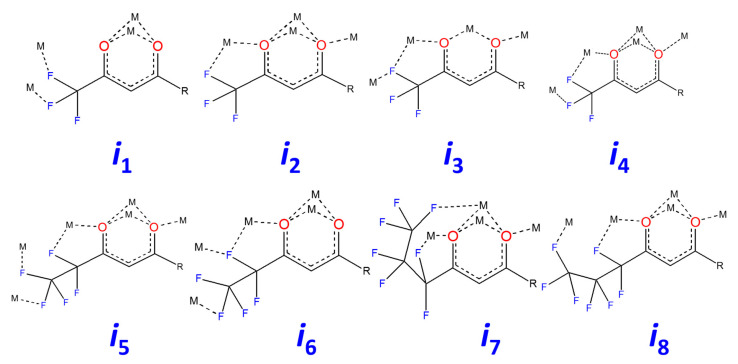
Coordination modes of the β-diketonate ligands in the structures **1**–**6**.

In turn, β-diketonate-ligands are able to perform various coordination functions that are distinguished by the involvement of oxygen atoms as chelating or chelating-bridging and the different number of fluorine atoms involved in the terminal contacts with alkali metal cations. The analysis of metal–ligand interactions revealed eight different coordination modes of β-diketonates in the studied complexes (Figure 2).

#### 2.2.1. Structure Description

**Figure 3:**
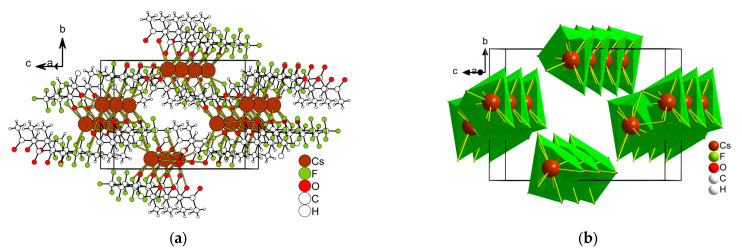
The fragment of the structure of Cs(Me..C_2_F_5_) **1** (**a**), the same fragment showing only Cs^+^ polyhedra (**b**).

*Cs(Me..C_2_F_5_)* **1** crystallizes in a monoclinic space group *P2**_1_**/n*, and its asymmetric unit contains one Cs(Me..C_2_F_5_) fragment (Figure 3). The Cs^+^ center is composed of nine atoms— six O atoms and three F atoms from five anions. The geometry around Cs^+^ exhibits a spherical-relaxed capped cube (CCU-9). The distances of the Cs–O bonds vary from 2.9909(18) to 3.1792(19) Å, while the distance of the Cs–F bond lengths is 3.2129(16)–3.376(16) Å. The coordination mode of the anion is i_5_ in Figure 2—an anion links five metal cations. The polyhedra are joined via the square face or edge, forming a 3D framework structure with the shortest distance between the cations being 3.369 Å.

**Figure 4:**
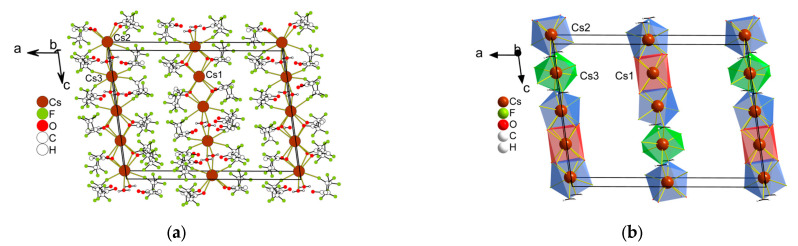
The fragment of the structure of Cs(Me..C_3_F_7_)(H_2_O) **2** (**a**), the same fragment showing only Cs^+^ polyhedra (**b**). Hydrogen atoms of water molecules are not omitted.

*Cs(Me..C_3_F_7_)(H_2_O)* **2** crystallizes in a monoclinic space group, *C2/c*, and its asymmetric unit contains three Cs^+^ (Cs1 and Cs2 are located on the second order axis), two anions, and two water molecules (Figure 4). The Cs1 centers are made up of eight O atoms from four anions, Cs2 (4O + 4F) from four anions, and Cs3 (6O + 2F) from four anions. The geometry around Cs1 and Cs2 exhibits a cube (CU-8) and for Cs3 it is a square antiprism (SAPR-8) or triangular dodecahedron (TDD-8). The distances of the Cs–O bonds vary from 3.083(3) to 3.256(3) Å, while the range of Cs–F bond lengths is 3.225(2)–3.391(3) Å. The coordination mode of the anion is i_3_ in Figure 2. An anion links four metal cations and the polyhedra are joined via the square face or edge, forming a 2D layered structure in the *bc* plane. Medium-strength hydrogen bonds between water molecules and anion oxygen atoms are realized in the layers (d(D∙∙∙A) = 2.745(2)–2.847(2) Å) (Supplementary Materials, Figure S2a). There are only van der Waals interactions between the layers F...H (≥2.940 Å), H...H (≥3.155 Å), and F...F (≥3.069 Å).

**Figure 5:**
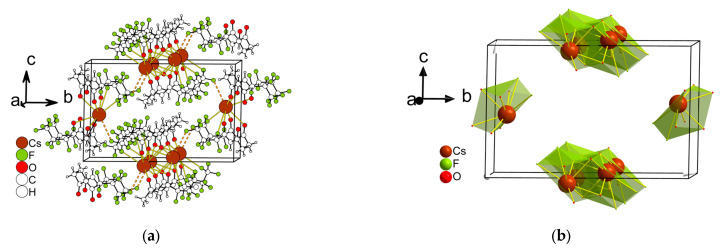
The fragment of the structure of Cs(Me..C_3_F_7_) **3** (**a**), the same fragment showing only Cs^+^ polyhedra (**b**). The elongated Cs–F contacts are not indicated.

*Cs(Me..C_3_F_7_)* **3** crystallizes in a monoclinic space group, *P2**_1_**/n*, and its asymmetric unit contains one [Cs(Me..C_3_F_7_)] fragment (Figure 5). The Cs^+^ center is composed of eight atoms—six O atoms and two F atoms from five anions. The geometry around Cs^+^ exhibits a heptagonal pyramid (HPY-8). The distances of the Cs–O bonds vary from 3.061(3) to 3.207(3) Å, while the range of Cs–F bond lengths is 3.273(3)–3.283(3) Å. The coordination environment of the cation is added with elongated contacts Cs–F 3.462, 3.627, and 3.673 Å that support the “open” polyhedron. The coordination mode of the anion is i_7_ in Figure 2. An anion links four metal cations. The polyhedra are joined via the square face or edge, forming chains along the *a* direction. These chains are connected into a 3D framework structure through a Cs–F contact of 3.462 Å (Figure 5a, dotted lines) that is stabilized by other elongated Cs–F contacts. The shortest distance between the cations is 3.947 Å.

**Figure 6:**
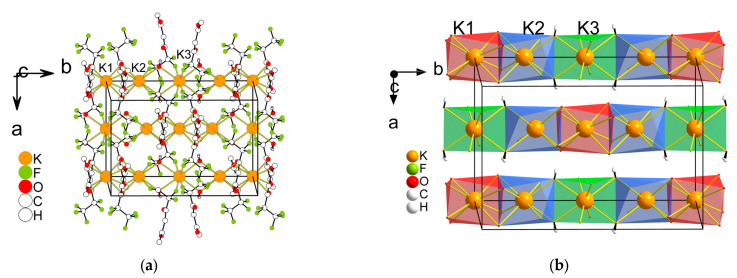
The fragment of the structure of K(Me..C_2_F_5_)(H_2_O) **4** (**a**), the same fragment showing only K^+^ polyhedra (**b**). Hydrogen atoms of water molecules are not omitted.

*K(Me..C_2_F_5_)(H_2_O)* **4** crystallizes in a monoclinic space group, *P*2_1_/*n*, and its asymmetric unit contains three K^+^ (all cations are located on the rotation axis), one anion, and one water molecule (Figure 6). The K1 centers are composed of eight O atoms from four anions, K2 (4O + 4F) from four anions, and K3 (6O + 2F) from four anions. The geometry around K1 and K2 exhibits a triangular dodecahedron (TDD-8), and for K3 it is a square antiprism (SAPR-8) or triangular dodecahedron (TDD-8). The K3 polyhedra are intermediate between a square antiprism (SAPR-8) and triangular dodecahedron (TDD-8). The distances of the K–O bonds vary from 2.7525(16) to 3.0343(17) Å, while the range of K–F bond lengths is 2.7144(14)–2.8980(15) Å. The coordination mode of the anion is i_1_ in Figure 2. The anion links four metal cations and the polyhedra are joined via square or triangle faces, forming a 2D layered structure in the *ab* plane. Medium-strength hydrogen bonds between water molecules and anion oxygen atoms are realized in the layers (d(D∙∙∙A) = 2.721–2.900 Å) (Supplementary Materials, Figure S2b). There are only van der Waals interactions between the layers F...H (≥3.056 Å), F...F (≥2.904 Å), and H...H (≥2.665 Å).

**Figure 7:**
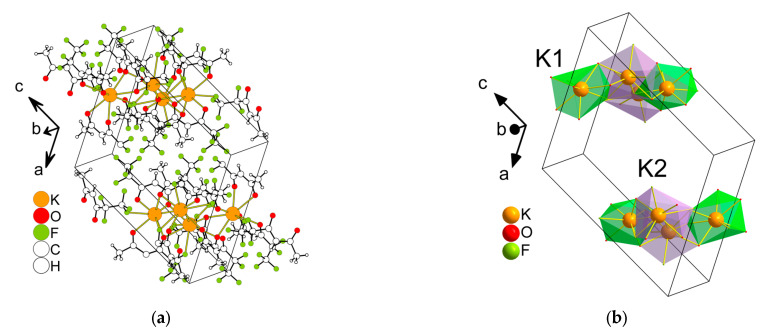
The fragment of the structure of K(Me..C_2_F_5_) **5** (**a**), the same fragment showing only K^+^ polyhedra (**b**).

*K(Me..C_2_F_5_)* **5** crystallizes in a monoclinic space group, *P2**_1_**/n*, and its asymmetric unit contains two [K(Me..C_2_F_5_)] fragments (Figure 7). The K1 centers are composed of six O atoms and two F atoms from four anions, and K2 (5O + 2F) from four anions. The geometry around K1 exhibits a triangular dodecahedron (TDD-8); for K2 it is capped trigonal prism (CTPR-7). The distances of the K–O bonds vary from 2.6631(13) to 2.8538(12) Å, while the range of K–F bond lengths is 2.7761(12)–3.0753(12) Å. The coordination modes of the anion are i_2_ and i_6_ in Figure 2. The anion links four and five metal cations. The polyhedra are joined via the square or triangle faces, forming the 2D layered structure in the (101) plane. There are only van der Waals interactions between the layers F...H (≥2.755 Å), F...F (≥3.046 Å), and H...H (≥3.155 Å).

**Figure 8:**
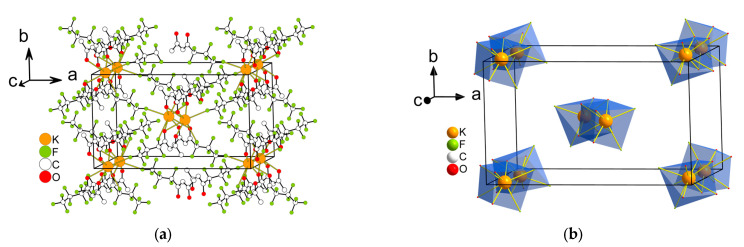
The fragment of the structure of K(Me..C_3_F_7_) **6** (**a**), the same fragment showing only K^+^ polyhedra (**b**).

*K(Me..C_3_F_7_)* **6** crystallizes in a monoclinic space group, *C*2/*c*, and its asymmetric unit contains one [K(Me..C_3_F_7_)] fragment (Figure 8). The K^+^ center contains six O atoms and two F atoms from five anions. The geometry around K^+^ exhibits a cube (CU-8). The distances of the K–O bonds vary from 2.9719(13) to 3.1046(11) Å, while the range of K–F bond lengths is 2.6429(12)–3.0468(14) Å. The coordination mode of the anion is i_8_ in Figure 2. An anion links five metal cations. The polyhedra are joined via the square face or edge, forming a 3D framework structure with the shortest distance between the cations being 3.505 Å.

#### 2.2.2. Structure Discussion

##### Features of Aqua-Complexes

The new aqua (hydrated) complexes K(Me..C_2_F_5_)(H_2_O) **4** and Cs(Me..C_3_F_7_)(H_2_O) **2** are both 2D coordination polymers. Moreover, one can note similar motifs in their structural organization. This includes the same coordination numbers and three types of cation environments (8O, 6O2F, and 4O4F) as well as the same sequence of their suits in the structure (8O → 6O2F → 4O4F → 6O2F → …, Figure 4 and Figure 6). Note that water molecules are only involved in the 4O4F type of environment and the polyhedra in this case are the same. However, the 8O and 6O2F types of environment are presented as CU-8 polyhedra for complex **2**, while for complex **4** they are TDD-8 polyhedra. The coordination modes of β-diketonate ligands are also different (*i*_3_ and *i*_1_, Figure 2), but in both structures, the anion links four metal cations, and these modes could be considered related (Figure S3a).

The comparison with the literature shows that the 2D layered structures are typical for aqua-β-diketonates of HAM (Table 1). The participation of water in the polyhedra of the 4O4F type is also a characteristic feature. In fact, a single exception is now known, namely, the K(Cl-Ph..CF_3_)(H_2_O) complex, where the anion contains a heteroatom [30] (Table 1). Note that the 8O environment formed by β-diketonate oxygen atoms is also typical [25,26]. Both types of the cation environment are predominantly presented as TDD-8 polyhedra (Table 1).

##### Features of Anhydrous Complexes

All anhydrous complexes under study are 3D coordination polymers, with the exception of K(Me..C_2_F_5_) **5** which forms a layered (2D) structure. The preferred type of cation environment is 6O2F. Two exceptions are observed in the *M*(Me..C_2_F_5_) structures and are associated with the change of the coordination number. For larger cesium cations, the additional F atom is included in the (6O3F) environment while for smaller potassium cations, one O atom departs from the nearest environment of one of the independent cations (5O2F).

In general, the comparison of β-diketonates with the same anion and different cations demonstrates the diversity of the structural organization. Thus, the same type of coordination environment (6O2F) is represented by different polyhedrals (CU-8, TDD-8, and HPY-8 for **3**, **5,** and **6**, respectively). The anion coordination modes are also different for all the structures. In *M*(Me..C_2_F_5_), the β-diketonate ligands link 5 and 4.5 (average) metal cations for cesium and potassium, respectively, which is consistent with their size. The opposite trend for *M*(Me..C_3_F_7_), i.e., the smaller number of bound cesium cations, is apparently due to the presence of additional extended Cs–F contacts. The relations between the anion coordination modes in the considered pairs of the complexes are shown in Figure S3.

The data on anhydrous compounds make it possible to compare the structure changes in the complete homologous series M(Me..R), R = CF_3_, C_2_F_5_, C_3_F_7_, but only for cesium. In the Cs(Me..CF_3_) structure [25], the anion coordination mode is i_4_ and is similar to i_5_ (Figure 2, the CF_3_ group plays a similar role as C_2_F_5_). The cation environment is typical (6O2F), but the polyhedron is in an equivocal form (TT-8, CU-8, or HBPY-8). The polyhedra join via square faces and edges, forming the layers in the *bc* plane.

In all three considered structures Cs(R..CH_3_), one can distinguish the “double chains” of cesium cations (Figure S4). For the first member of the homologous series (R = CF_3_), these chains are connected by anions into layers (Figure S4a), while, with an increase in the volume of the fluorinated substituent, they connect into a framework (Figure S4b,c). It is convenient to describe this by considering the β-diketonates coordination modes: in Cs(Me..CF_3_), all five cations belong to a single layer (i_4_ mode, Figure 2), while in Cs(Me..C_2_F_5_), four cations connected with O atoms belong to a layer and two cations connected only with terminal F atoms are outside a layer (i_5_ mode, Figure 2). In Cs(Me..C_3_F_7_), all four cations connected with O atoms belong to a layer (i_7_ mode, Figure 2), and the framework is formed by slightly elongated Cs–F_CF3_ terminal contacts (Figure 5a, dotted lines). Note that the top view of the discussed “double chains” in both framework structures shows that there is a hexagonal cation packing motif (Figure S5).

Unlike cesium, the potassium complex K(Me..C_2_F_5_) still has a layered structure, not a framework. However, the subsequent increase in the fluorinated group (C_2_F_5_ => C_3_F_7_) leads to the formation of a framework complex K(Me..C_3_F_7_). In this case, the “double chains” of cations similar to the cesium analogs could be also distinguished (Figure S6a) and these chains are hexagonally packed (Figure S6b). Concerning the ligand coordination mode in K(Me..C_3_F_7_), the framework is also formed by cations connected only with terminal F atom, while other cations belong to a layer of “double chains” (i_5_ mode, Figure 2).

The comparison of the pairs of hydrated and anhydrous complexes (**2** and **3**/**4** and **5**) shows that in the absence of coordinated water, the denticity of the β-diketonate ligand increases as expected. In particular, for potassium compounds K(Me..C_2_F_5_) **4** and **5**, the denticity is six and eight, respectively, and for cesium compounds Cs(Me..C_3_F_7_) **2** and **3**, the denticity is five and eight, respectively.

### 2.3. Thermal Properties

**Figure 9:**
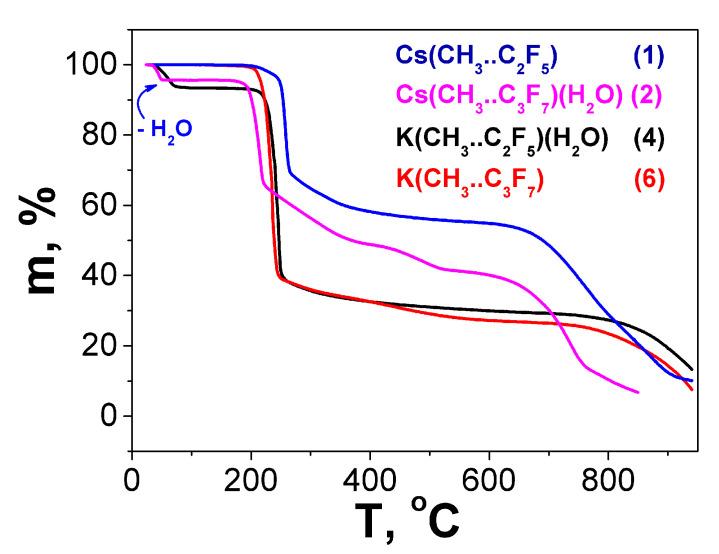
Thermal analysis data for Cs(Me..C_2_F_5_) (**1**), Cs(Me..C_3_F_7_)(H_2_O) (**2**), K(Me..C_2_F_5_)(H_2_O) (**4**), and K(Me..C_3_F_7_) (**6**).

The thermal properties of compounds **1**, **2**, **4**, and **6** were studied by thermogravimetry (Figure 9) at atmospheric pressure in an inert gas (He) flow and at a constant heating rate (10°/min) as in [25]. The obtained results are presented in Table 2.

It should be emphasized that the dehydration of all the investigated hydrated complexes starts at room temperature (Figure 9, Table 2, column 2). This is consistent with the lability observed during the synthesis and the inclusion of water in the compounds. This seems to be a feature of fluorinated HAM β-diketonates with alkyl substituents. In fact, in the presence of a phenyl substituent in the fluorinated ligand [25,26], as well as in the non-fluorinated ligand [28], dehydration occurs at a notably higher temperature range of 55–65 °C and 90 °C, respectively.

All the anhydrous β-diketonates were stable over a wide temperature range. The endo effects on the DTA curves show the melting process at 195 °C for Cs(Me..C_2_F_5_), 162 °C for Cs(Me..C_3_F_7_), 180 °C for K(Me..C_2_F_5_), and 172 °C for K(Me..C_3_F_7_). For potassium complexes, the decomposition temperatures are close, namely, 195 °C and 200 °C for **4** and **6**, respectively. This correlates with the similarity of the anion coordination modes that link 4.5 (average) and 5 metal cations, respectively.

For cesium complexes, the similarity of the TGA conditions with the previous study allowed us to construct the following stability row, based on decomposition temperatures:

Cs(Me..C_3_F_7_) (170 °C) ~ Cs(Me..CF_3_) (175 °C) [25] < Cs(Me..C_2_F_5_) (195 °C) < Cs(CF_3_..CF_3_) (220 °C) [25] < Cs(Ph..CF_3_) (240 °C) [25].

Within a homological series, Cs(Me..R), R = CF_3_, C_2_F_5_, C_3_F_7_, a nonmonotonic character in the change in the decomposition temperatures was observed. The decrease in the stability of the complex with the largest fluorinated substituent (**3** compared with **1**) may be a consequence of the weakening of bonding, since the anions in these structures link four and five cations, respectively.

The presence of several steps on the weight loss curves of the studied complexes indicates the complicated character of their decomposition, though the final decomposition products are amorphous. However, the main mass loss occurred at temperatures up to 300 °C (Figure 9). It should be noted that for both potassium complexes, the mass loss curves after 600 °C degrees are similar (Figure 9). Thus, the same main decomposition product can be assumed. This is also observed for a pair of cesium complexes (Figure 9).

According to the calculations from the mass loss curves, the mass residues on the plateau are more consistent with metal carbonates than fluorides (Table 2). However, the formation of the metal fluorides from fluorinated β-diketonate complexes is more typical [24,35,36]. In order to testify to the decomposition product, we investigated the thermal behavior of KF 2H_2_O under the same conditions (Supplementary Materials, Figure S7). Indeed, in the high-temperature region, the mass loss curves of the KF and potassium complexes were similar, and the mass loss above 800 °C seems to be due to fluoride evaporation. This primarily confirms the formation of metal fluorides. Exceeding the calculated mass (Table 2), in this case, can be associated with the presence of carbon products from the ligand decomposition.

## 3. Materials and Methods

### 3.1. Materials and Methods

All chemicals were commercially available products of reagent grade and used without further purification.

Elemental analysis was carried out in the Multi-Access Chemical Research Center of the Siberian Branch of the Russian Academy of Sciences using an EURO EA 3000 automatic CHNS analyzer (EuroVector, Pavia, Italy) for carbon and hydrogen determination and a Cary-50 spectrophotometer for fluorine determination [37,38]. The accuracies were no more than 0.5% (mass).

IR spectra were recorded for KBr pellets (or fluorinated oil suspensions) using a Scimitar FTS 2000 spectrometer (Digilab LLC, Canton, USA) in the 400–4000 cm^−1^ wavenumber range.

X-ray powder diffraction (PXRD) measurements were performed on a Shimadzu XRD-7000 diffractometer (Shimadzu, Kyoto, Japan) (Cu*K*α radiation, Ni filter, linear One Sight detector, 2θ = 5–60°).

Thermal analysis was carried out on a Netzsch TG 209 F1 Iris thermal analyzer (NETZSCH-geratebau GmbH, Selb, Germany). The sample weight was 10 ± 1 mg and the experiments were carried out in a helium atmosphere (30.0 mL/min, Al_2_O_3_ open crucible, 10 °C/min).

### 3.2. Synthesis

Potassium and cesium complexes were synthesized by the reaction of M_2_CO_3_, (M = K, Cs) with β-diketone in ethanol (EtOH). The reaction mixture was stirred for 4 h at room temperature (RT). The product was separated after the evaporation of the solution, dried in the air, and purified by reprecipitation. To this end, the product was dissolved in acetone. After complete dissolution, the obtained solution was heated (~50 °C), and cold (~−10 °C) hexane was added to it in a five-fold excess. The obtained fine precipitate was filtered off.

*Cs(Me..C_2_F_5_)* (**1**) was obtained from 0.24 g of Cs_2_CO_3_ (0.74  mmol) and 0.32  g of HL (1.54  mmol) in 10  mL of EtOH. The solution was evaporated, yielding 0.48 g of **1** (1.42  mmol), with a yield of 95%. Anal Calcd. for C_6_H_4_F_5_O_2_Cs (mass. %): C, 21.5; H, 1.2; and F, 28.3. We found: C, 21.9; H, 1.9; and F, 28.3. IR spectrum (KBr, ν, cm^−1^): 1656, 1536, 1497, and 1428 (s) (ν(C = O) + ν(C = C)); 1354, 1328 (w) (δ(C-H)); 1216, 1188, 1163, 1119, and 1026 (ν(CF)).

*Cs(Me..C_3_F_7_)(H_2_O)* (**2**) was obtained from 0.21 g of Cs_2_CO_3_ (0.65 mmol) and 0.36  g of HL (0.32  mmol) in 10  mL of EtOH. The solution was evaporated, yielding 0.39  g of **2** (1.04  mmol), and a yield of 80%. Anal Calcd. for C_7_H_8_F_7_O_4_Cs (CsL(H_2_O)_2_) (mass. %): C, 19.9; H, 1.9; and F, 31.5. We found: C, 20.3; H, 1.5; and F, 31.8. IR spectrum (KBr, ν, cm^−1^): 3444 (m) (ν(OH)); 1632, 1496, and 1472 (s) (ν(C = O) + ν(C = C)); 1349 (w) (δ(C-H)); 1231, 1153, and 1107 (s) (ν(CF)).

*Cs(Me..C_3_F_7_)* (**3**) was obtained by keeping **2** in P_2_O_5_ desiccators for two weeks. IR spectrum (fluorinated oil, ν, cm^−1^): 2960, 2922, and 2853 (w) (ν(CH)); 1641, 1486 (s) (ν(C = O) + ν(C = C)).

*K(Me..C_2_F_5_)(H_2_O)* (**4**) was obtained from 0.17  g of K_2_CO_3_∙1.5H_2_O (1.03  mmol) and 0.43 g of HL (2.10  mmol) in 10 mL of  EtOH. The solution was evaporated, yielding 0.42  g of **4** (1.75  mmol), and a yield of 85%. Anal Calcd. for C_6_H_4_F_5_O_2_K (KL) (mass %): C, 29.8; H, 1.7; and F, 39.2. Anal Calcd. for C_6_H_6_F_5_O_3_K (KL(H_2_O)) (mass %): C, 27.7; H, 2.3; and F, 36.5. We found: C, 29.5; H, 1.7; and F, 36.1. IR spectrum (KBr, ν, cm^−1^): 3483, 3273 (m) (ν(OH)); 1705, 1655, 1528, and 1428 (s) (ν(C = O) + ν(C = C)); 1355, 1328 (w) (δ(C-H)); 1237, 1213, 1179, 1160, and 1118 (s) (ν(CF)).

*K(Me..C_2_F_5_)* (**5**) was obtained by keeping **4** in P_2_O_5_ desiccators for two weeks. IR spectrum (fluorinated oil, ν, cm^−1^): 2994, 2929, and 2852 (w) (ν(CH)); 1642, 1530, and 1503 (s) (ν(C = O) + ν(C = C)).

*K(Me..C_3_F_7_)* (**6**) was obtained from 0.14 g of K_2_CO_3_∙1.5H_2_O (0.86  mmol) and 0.45 g of HL (1.76  mmol) in 10 mL of  EtOH. The solution was evaporated, yielding 0.48  g of **6** (1.62  mmol), and a yield of 95%. Anal Calcd. for C_7_H_4_F_7_O_2_K (mass %): C, 28.8; H, 1.4; and F, 45.5. Anal Calcd. for C_14_H_10_F_14_O_5_K_2_ (K_2_(L)_2_(H_2_O)) (mass %): C, 27.9; H, 1.7; and F, 44.2. We found: C, 27.7; H, 1.5; and F, 45.0. IR spectrum (KBr, ν, cm^−1^): 1643, 1528, 1501, and 1438 (s) (ν(C = O) + ν(C = C)); 1354 (w) (δ(C-H)); 1273, 1230, 1206, 1180, 1155, 1126, and 1111(s) (ν(CF)).

### 3.3. Structural Investigation

The crystals suitable for single-crystal X-ray diffraction analysis (SXRD) were obtained by a slow evaporation technique with a single solvent for **4** (Et_2_O) and **6** (EtOAc), and mixture solvents were used for **1** (EtOAc/hexane). The **2** crystals were captured by liquid–liquid diffusion when the solution of the complex in diethyl ether was carefully layered with hexane. Other crystals, **3** and **5,** were obtained by slow extraction with Et_2_O in a vacuum-sealed L-shaped ampoule with two knees [39].

The SXRD experiments for **1**–**6** were performed on a Bruker Nonius X8 Apex 4 K CCD diffractometer fitted with graphite monochromated MoKα radiation. The data were collected at 150  K or 220 K for all the structures by standard technique [40]. Absorption corrections were made empirically using SADABS. The structures were solved by direct methods and further refined by the full-matrix least squares model using the SHELXTL program package [41]. Hydrogen positions of β-diketonate ligand were calculated geometrically and refined in the rigid body approximation (riding model). The hydrogen positions of the water molecules were refined with a fixed O–H bond length (0.956 ± 0.002 Å [42]) in the isotropic approximation. The structures were deposited in the Cambridge Crystallographic Data Centre (CCDC) with the reference codes 2282726–2282731 (www.ccdc.cam.ac.uk/data_reguest/cif (accessed on 18 July 2023)).

## 4. Conclusions

We report the synthesis and detailed characterization of novel potassium and cesium complexes with (Me..C_2_F_5_) and (Me..C_3_F_7_) β-diketonate ligands. Such compounds can differ in the presence of water in their structure, and a simple and convenient method for the preparation of anhydrous fluorinated alkali metal β-diketonates was proposed herein.

Summarizing the structural information, we showed that the layered structure motif is the most typical for heavy alkali metal β-diketonate complexes. In fact, to the best of our knowledge, no examples of hydrated compounds of this class with framework structures are yet known. For anhydrous β-diketonates, increasing the size of the fluorinated group in the (Me..R)^−^ anion led to the formation of coordination frameworks: starting from R = C_2_F_5_ for cesium and from C_3_F_7_ for potassium. Variations in the coordination modes of the β-diketonate ions in these compounds are highly diverse, and for cations, CN = 7–8 (K^+^) and 8–9 (Cs^+^) are realized.

The TGA data provide insight into the lability of the incorporation/withdrawal of water in the complexes. Most importantly, an increase in the length of the fluorinated substituent in the HAM β-diketonate complexes leads to a decrease in the melting temperature (Δ = 8–33°), and for cesium compounds, a significant decrease in their thermal stability (Δ = 25°). This feature could be beneficial to produce fluorinated perovskites via spin-coating and thermal approaches.

## Figures and Tables

**Table 1:** The nearest environment of cations in the structures of potassium and cesium β-diketonates.

Compound	Coordination Number	Environment	Polyhedra	Reference
Cs(Me..C_2_F_5_) **1**	9	6O3F	CCU-9	This work
Cs(Me..C_3_F_7_)(H_2_O) **2**	8	6O2F	CU-8	
	8	8O	CU-8	
	8	4O4F	SAPR-8 or TDD-8	
Cs(Me..C_3_F_7_) **3**	8	6O2F	HPY-8	
K(Me..C_2_F_5_)(H_2_O) **4**	8	8O	TDD-8	
	8	6O2F	TDD-8	
	8	4O4F	SAPR-8 or TDD-8	
K(Me..C_2_F_5_) **5**	8	6O2F	TDD-8	
	7	5O2F	CTPR-7	
K(Me..C_3_F_7_) **6**	8	6O2F	CU-8	
K(Ph..CF_3_)(H_2_O)	8	8O	TDD-8	[26]
	8	4O4F	TDD-8	
K(Cl-Ph..CF_3_)(H_2_O)	8	6O2F	CU-8	[30]
Cs(Ph..CF_3_)(H_2_O)	8	8O	TDD-8	[25]
	8	4O4F	TDD-8	
Cs(Me..CF_3_)	8	6O2F	TT-8 or CU-8 or HBPY-8	[25]

Reference shape: HPY-8—heptagonal pyramid (C_7v_), CU-8—cube (O_h_), SAPR-8—square antiprism (D_4d_), TDD-8—triangular dodecahedron (D_2d_), HBPY-8—hexagonal bipyramid (D_6h_), TT-8—triakis tetrahedron (T_d_), CCU-9—spherical-relaxed capped cube (C_4v_), and CTPR-7—capped trigonal prism (C_2v_).

**Table 2:** The results of thermal analysis for Cs(Me..C_2_F_5_) (**1**), Cs(Me..C_3_F_7_)(H_2_O) (**2**), K(Me..C_2_F_5_)(H_2_O) (**4**), and K(Me..C_3_F_7_) (**6**).

Compound	*T*_water loss_	Water Loss Exp.(Calc.), Mass.%	T_decomp._	Decomposition Products (Residues) Exp.(Calc.), Mass.%
Cs(Me..C_2_F_5_) **1**	–	–	195 °C	57.0 (42.7, CsF/48.5, ½ Cs_2_CO_3_)
Cs(Me..C_3_F_7_)(H_2_O) **2**	25–61 °C	5.0 (4.5, -H_2_O)	170 °C	41.4 (37.6, CsF/40.3, ½ Cs_2_CO_3_)
K(Me..C_2_F_5_)(H_2_O) **4**	25–82 °C	6.4 (6.9, -H_2_O)	200 °C	28.9 (22.3, KF/26.6 ½ K_2_CO_3_)
K(Me..C_3_F_7_) **6**	–	–	195 °C	26.7 (19.9, KF/23.7 ½ K_2_CO_3_)

## Data Availability

The data presented in this study are available on request from the corresponding author. The CIF files containing full crystallographic data for **1**–**6** (CCDC 2282726–2282731) can be obtained free of charge via https://www.ccdc.cam.ac.uk/structures (accessed on 18 July 2023).

## Supplementary Materials

The following supporting information can be downloaded at: https://www.mdpi.com/article/10.3390/molecules28155886/s1, Figure S1: Experimental (bottom black) and calculated (top red) powder XRD patterns of **1**–**6**; Figure S2: The fragment of the structure of *Cs(Me..C_3_F_7_)(H_2_O)* (a) and *K(Me..C_2_F_5_)(H_2_O)* (b); Figure S3: Relationships between the coordination modes of β-diketonate ligands; Figure S4: The fragments of the structure of *Cs(Me..CF_3_)* (a), *Cs(Me..C_2_F_5_)* (b), and *Cs(Me..C_3_F_7_)* (c); Figure S5: Hexagonal motif of cesium cation packing in the structures *Cs(Me..C_2_F_5_)* (a) and *Cs(Me..C_3_F_7_)* (b); Figure S6: The fragment of “double chains” (a) and hexagonal motif of potassium cation packing (b) in the structure of *K(Me..C_3_F_7_)*; Figure S7: Thermal analysis data for *KF(H_2_O)_2_*; Table S1: Crystallographic data and experimental conditions for complexes **1**–**6**; Table S2: Polyhedra analysis for potassium and cesium β-diketonate.

## Sample Availability

Samples of compounds **1**–**6** are available from the authors.

## References

[1] Isayama S., Mukaiyama T. (1989). Hydration of Olefins with Molecular Oxygen and Triethylsilane Catalyzed by Bis(trifluoroacetylacetonato)cobalt(II). Chem. Lett..

[2] Binnemans K. (2009). Lanthanide-based luminescent hybrid materials. Chem. Rev..

[3] Nehra K., Dalal A., Hooda A., Bhagwan S., Saini R.K., Mari B., Kumar S., Singh D. (2022). Lanthanides β-diketonate complexes as energy-efficient emissive materials: A review. J. Mol. Struct..

[4] Mishra S., Daniele S. (2015). Metal-organic derivatives with fluorinated ligands as precursors for inorganic nanomaterials. Chem. Rev..

[5] Dar W.A., Ganaie A.B., Iftikhar K. (2018). Synthesis and photoluminescence study of two new complexes [Sm(hfaa)_3_(impy)_2_] and [Eu(hfaa)_3_(impy)_2_] and their PMMA based hybrid films. J. Lumin..

[6] DuChane C.M., Merola J.S. (2020). Hexafluoroacetylacetonate (hfac) as ligand for pentamethylcyclopentadienyl (Cp*) rhodium and iridium complexes: Some surprising results, including an Ir_3_Na_1_O_4_ cubane structure. J. Organomet. Chem..

[7] Kudyakova Y.S., Slepukhin P.A., Valova M.S., Burgart Y.V., Saloutin V.I., Bazhin D.N. (2019). The impact of alkali metal ion on the crystal structure and (mechano)luminescence of terbium(III) tetrakis(β-diketonates). Eur. J. Inorg. Chem..

[8] Devi A. (2013). ‘Old Chemistries’ for new applications: Perspectives for development of precursors for MOCVD and ALD applications. Coord. Chem. Rev..

[9] Saloutin V.I., Edilova Y.O., Kudyakova Y.S., Burgart Y.V., Bazhin D.N. (2022). Heterometallic Molecular Architectures Based on Fluorinated β-Diketone Ligands. Molecules.

[10] Salmankurt B., Duman S. (2016). Investigation of the structural, mechanical, dynamical and thermal properties of CsCaF_3_ and CsCdF_3_. Mater. Res. Express..

[11] Raja A., Nagaraj R., Ramachandran K., Sivasubramani V., Annadurai G., Joseph D.D., Ramasamy P. (2020). A novel bifunctional Dy^3+^ activated RbCaF_3_ single phase phosphor: Facile synthesis and dual-luminescence properties for WLEDs and dosimetry applications. Adv Powder Technol..

[12] Quan Z., Yang P., Li C., Yang J., Yang D., Jin Y., Lian H., Li H., Lin J. (2009). Shape and Phase-Controlled Synthesis of KMgF_3_ Colloidal Nanocrystals via Microwave Irradiation. J. Phys. Chem. C..

[13] Hua R.N., Yu J.C., Jiang H., Shi C. (2007). Solvothermal synthesis and luminescent properties of the complex fluorides KMgF_3_: Eu and KZnF_3_:RE (RE = Eu, Ce). J. Alloys Compd..

[14] Sommerdijk J.L., Bril A. (1976). On the position of the ^5^D_0_ level of Eu^3+^ in AMgF_3_ (A = K, Rb, Cs). J. Lumin..

[15] Alcala R., Sardar D.K., Sibley W.A. (1982). Optical transitions of Eu^2+^ IONS in RbMgF_3_ crystals. J. Lumin..

[16] Wang J., Wang F., Wang C., Liu Z., Liu X.G. (2011). Single-band Upconversion Emission in Lanthanide-Doped KMnF_3_ Nanocrystals. Angew. Chem. Int. Ed..

[17] Lopez Ortiz J.C., Guerra G.A.F., Machado F.L.A., Rezende S.M. (2014). Magnetic Anisotropy of Antiferromagnetic RbMnF_3_. Phys. Rev. B Condens. Matter Mater. Phys..

[18] Seavey M.H. (1969). Nuclear and Electronic Spin-Wave Relaxation Rates in the Hexagonal Antiferromagnet CsMnF_3_. J. Appl. Phys..

[19] Pellegrino A.L., Lo Presti F., Malandrino G. (2022). A molecular route to fluoro-perovskite materials: Synthesis of CsCaF_3_ films through a sol-gel/spin-coating process. Discov. Mater..

[20] Park H.H., Senegas J., Reau J.M., Pezat M., Darriet B., Hagenmuller P. (1988). The CsCaF_3-x_H_x_ solid solution (0 ≤ x ≤ 1.70): Structural characteristics and hydrogen diffusion investigation. Mat Res Bull..

[21] Usman M., Ayer G.B., Smith M.D., zur Loye H.-C. (2021). Synthesis and Crystal Structure of a 6H Hexagonal Fluoro-Perovskite: RbMgF_3_. J. Chem. Crystallogr..

[22] Okazaki A., Suemune Y. (1961). The Crystal Structures of KMnF_3_, KFeF_3_, KCoF_3_, KNiF_3_ and KCuF_3_ above and below their Néel Temperatures. J. Phys. Soc. Jpn..

[23] Pellegrino A.L., Malandrino G. (2021). Surfactant-free synthesis of the full inorganic perovskite CsPbBr_3_: Evolution and phase stability of CsPbBr_3_ vs. CsPb_2_Br_5_ and their photocatalytic properties. ACS Appl. Energy Mater..

[24] Peddagopu N., Sanzaro S., Rossi P., Paoli P., Malandrino G. (2021). A one-pot synthesis of “K(hfa) glyme” adducts: Effect of the polyether length on the ion coordination sphere. Eur. J. Inorg. Chem..

[25] Vikulova E.S., Zherikova K.V., Kuratieva N.V., Morozova N.B., Igumenov I.K. (2013). Synthesis, structure, and thermal properties of fluorinated cesium beta-diketonates. J. Coord. Chem..

[26] Kochelakov D.V., Vikulova E.S., Kuratieva N.V. (2020). Potassium and Rubidium Benzoyltrifluoroacetonates: A Crystal Chemical Study and Thermal Properties. J. Struct. Chem..

[27] Kochelakov D.V., Vikulova E.S., Kuratieva N.V., Sukhikh A.S., Gromilov S.A. (2023). Study of potassium, rubidium hexafluoroacetylacetonates and by-products of their synthesis and crystallization. J. Struct. Chem..

[28] Tsymbarenko D.M., Korsakov I.E., Lyssenko K.A., Troyanov S.I. (2015). One-dimensional coordination polymers in the crystal structures of sodium and potassium acetylacetonates. Polyhedron.

[29] Rizvi M.A., Ali A., Iqbal M.S. (2007). Synergistic extraction of Ce(III), Tb(III) and Lu(III) with a mixture of hexafluoroacetylacetone and triphenylphosphineoxide in benzene. Radiochim. Acta.

[30] Martins J.P., Arranja C.C., Sobral A.J.F.N., Ramos Silva M. (2013). Poly[μ2-aqua-μ4-[1-(4-chlorophenyl)-4,4, 4-trifluorobutane-1,3-dionato]-potassium]. Acta Crystallogr. Sect. E Struct. Rep. Online.

[31] Gagné O.C., Hawthorne F.C. (2016). Bond-length distributions for ions bonded to oxygen: Alkali and alkaline-earth metals. Acta Crystallogr. Sect. B Struct. Sci. Cryst. Eng. Mater..

[32] Casanova D., Llunell M., Alemany P., Alvarez S. (2005). The Rich Stereochemistry of Eight-Vertex Polyhedra: A Continuous Shape Measures Study. Chem. Eur. J..

[33] Grebenyuk D., Zobel M., Polentarutti M., Ungur L., Kendin M., Zakharov K., Degtyarenko P., Vasiliev A., Tsymbarenko D. (2021). A Family of Lanthanide Hydroxo Carboxylates with 1D Polymeric Topology and Ln4 Butterfly Core Exhibits Switchable Supramolecular Arrangement. Inorg. Chem..

[34] Kendin M., Tsymbarenko D. (2020). 2D-Coordination Polymers Based on Rare-Earth Propionates of Layered Topology Demonstrate Polytypism and Controllable Single-Crystal-to-Single-Crystal Phase Transitions. Cryst. Growth Des..

[35] Peddagopu N., Rossi P., Bonaccorso C., Bartasyte A., Paoli P., Malandrino G. (2020). Facile synthesis of novel lithium β-diketonate glyme adducts: The effect of molecular engineering on the thermal properties. Dalton Trans..

[36] Peddagopu N., Pellegrino A.L., Bonaccorso C., Rossi P., Paoli P., Malandrino G. (2022). Sodium β-Diketonate Glyme Adducts as Precursors for Fluoride Phases: Synthesis, Characterization and Functional Validation. Molecules.

[37] Mikhailovskaya T.F., Makarov A.G., Selikhova N.Y., Makarov A.Y., Pritchina E.A., Bagryanskaya I.Y., Vorontsova E.V., Ivanov I.G., Tikhova V.D., Gritsan N.P. (2016). Carbocyclic functionalization of quinoxalines, their chalcogen congeners 2,1,3-benzothia/selenadiazoles, and related 1,2-diaminobenzenes based on nucleophilic substitution of fluorine. J. Fluor. Chem..

[38] Tikhova V.D., Fadeeva V.P., Nikulicheva O.N., Dobinskaya T.A., Deryabina Y.M. (2022). Determination of fluorine in organic functional materials. Chem. Sust. Develop..

[39] Li T., Gamer M.T., Scheer M., Konchenko S.N., Roesky P.W. (2013). Intramolecular Phosphorus–Phosphorus Bond Formation within a Co_2_P_4_ Core. Chem. Comm..

[40] Bruker Advanced X-ray Solutions (2004). APEX2 (Version 1.08), SAINT (Version 7.03), and SADABS (Version 2.11).

[41] Sheldrick G.M. (2015). IUCr Crystal Structure Refinement with SHELXL. Acta Crystallogr. Sect. C Struct. Chem..

[42] Ferraris G., Franchini-Angela M. (1972). Survey of the geometry and environment of water molecules in crystalline hydrates studied by neutron diffraction. Acta Crystallogr. Sect. B Struct. Crystallogr. Cryst. Chem..

