# Supplementary material for: Potassium and Cesium Fluorinated β-Diketonates: Effect of a Cation and Terminal Substituent on Structural and Thermal Properties
Source: Molecules. 2023 Aug 4;28(15):5886. doi: 10.3390/molecules28155886 (PMC10421003; doi:10.3390/molecules28155886)
Supplement: Supplementary file 1 [file molecules-28-05886-s001.zip › molecules-2540440-supplementary.pdf]

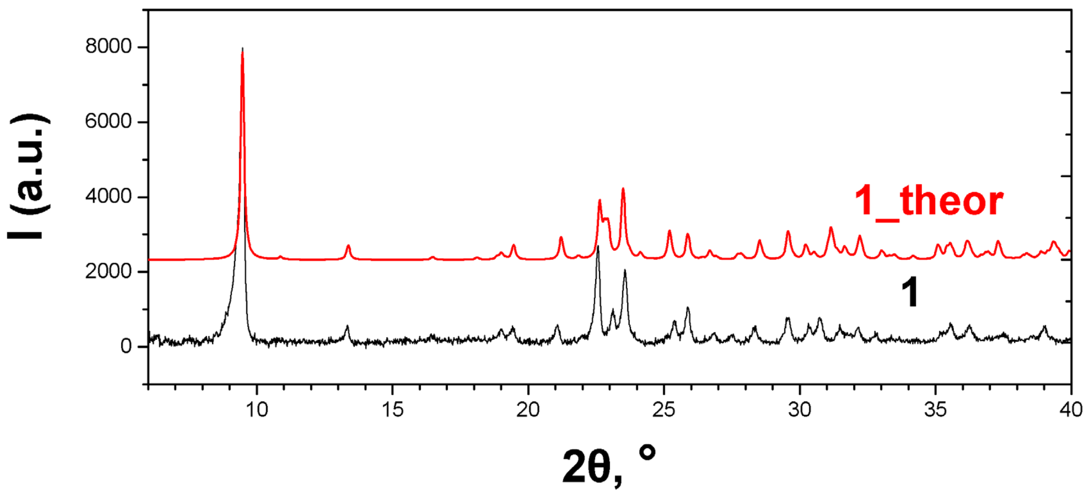
I (a.u.)
1_theor
1
2θ, °

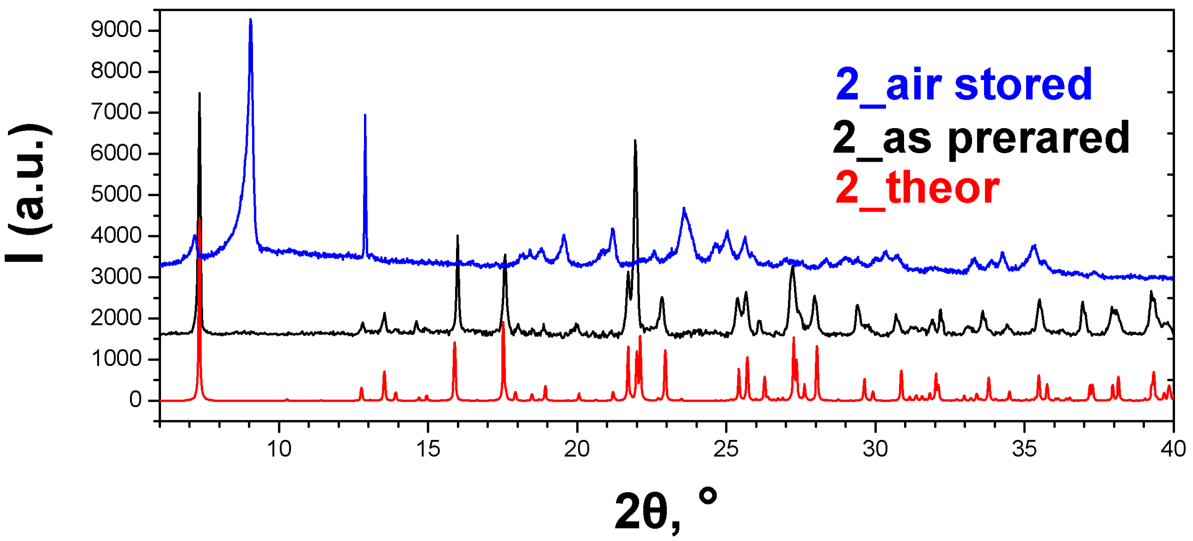
I (a.u.)
2_air stored
2_as prerared
2_theor
2θ, °

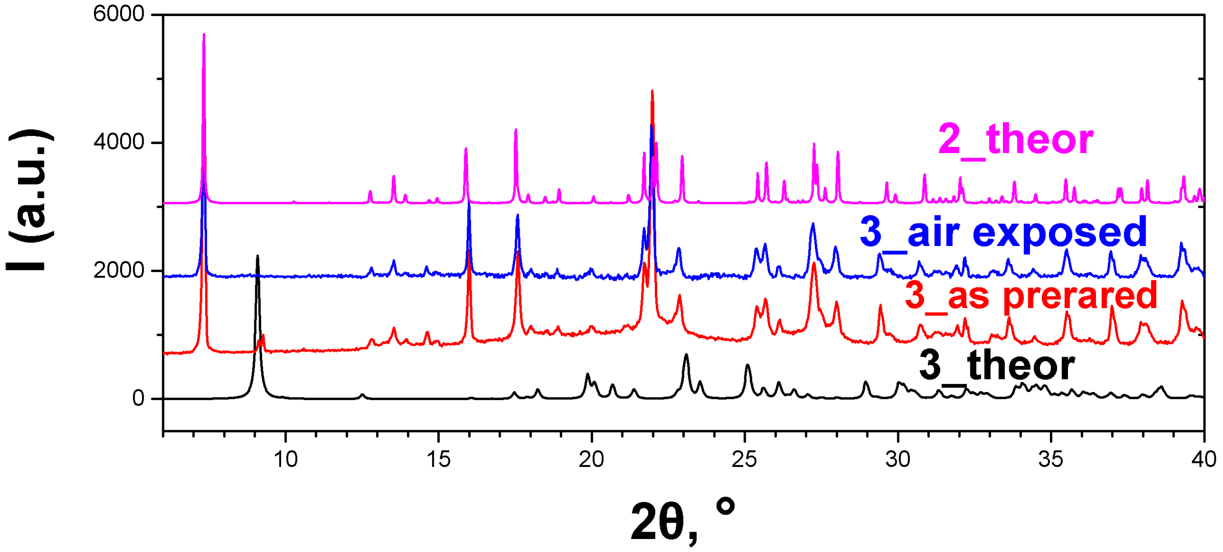
I (a.u.)
2_theor
3_air exposed
3_as prerared
3_theor
2θ, °

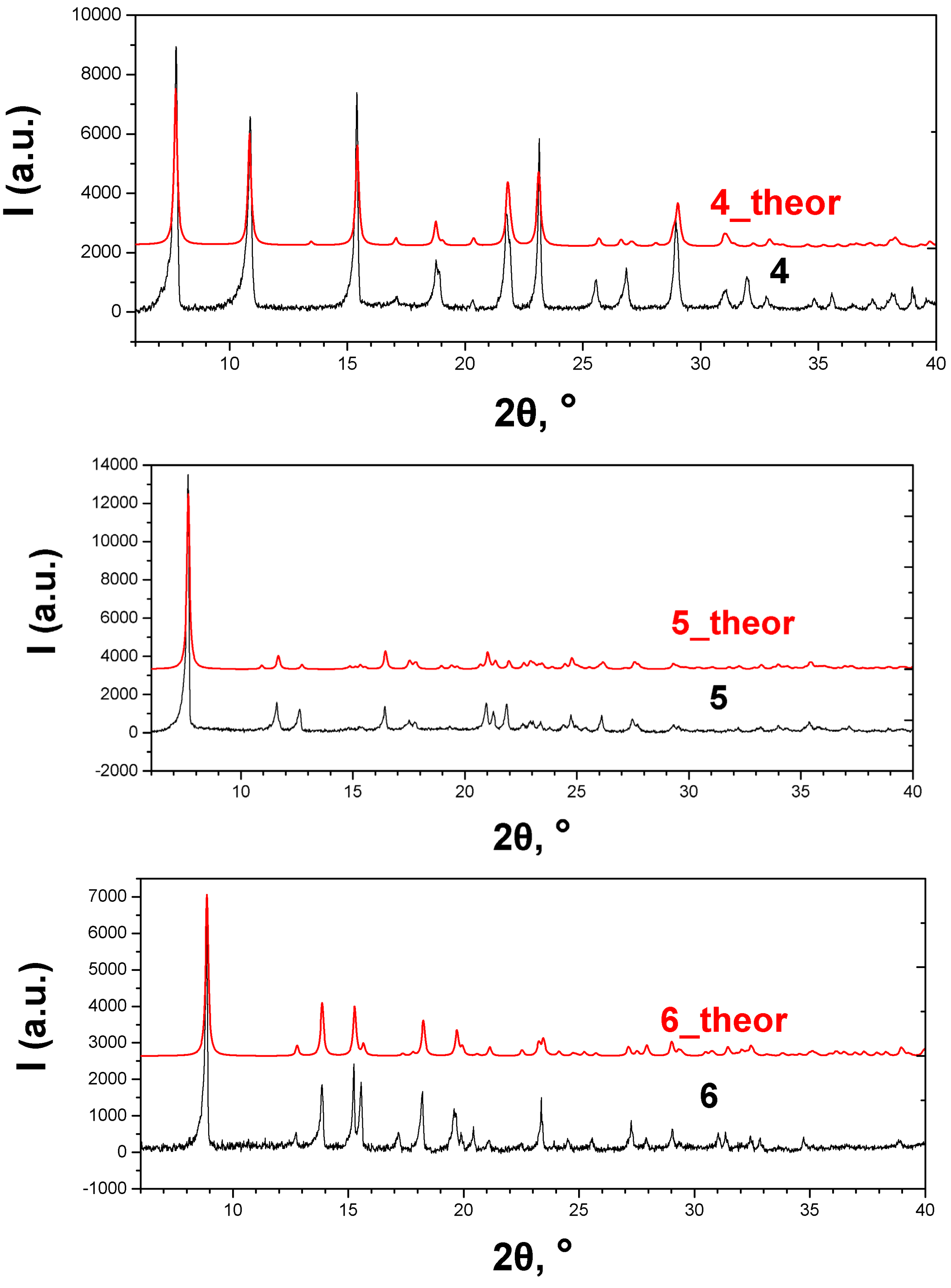

Figure S1. Experimental (bottom black) and calculated (top red) powder XRD patterns of **1-6**.

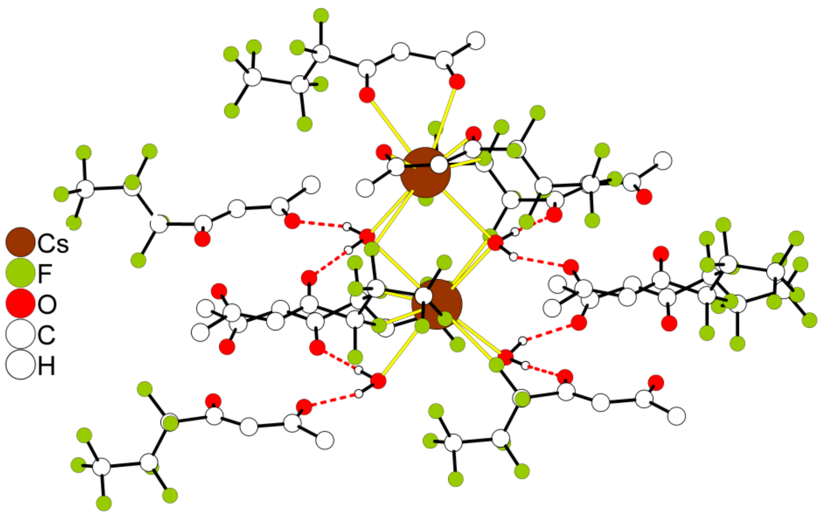

a

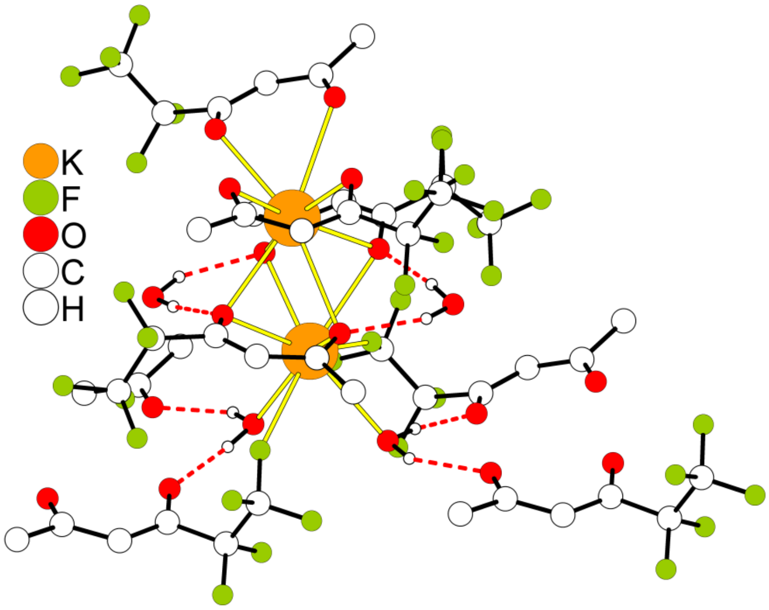

b

Figure S2. The fragments of the structures of *Cs(Me..C*$_3$*F*$_7$*)(H*$_2$*O)* (a) and *K(Me..C*$_2$*F*$_5$*)(H*$_2$*O)* (b) showing hydrogen bonds between water molecules and anion oxygen atoms (red dotted line).

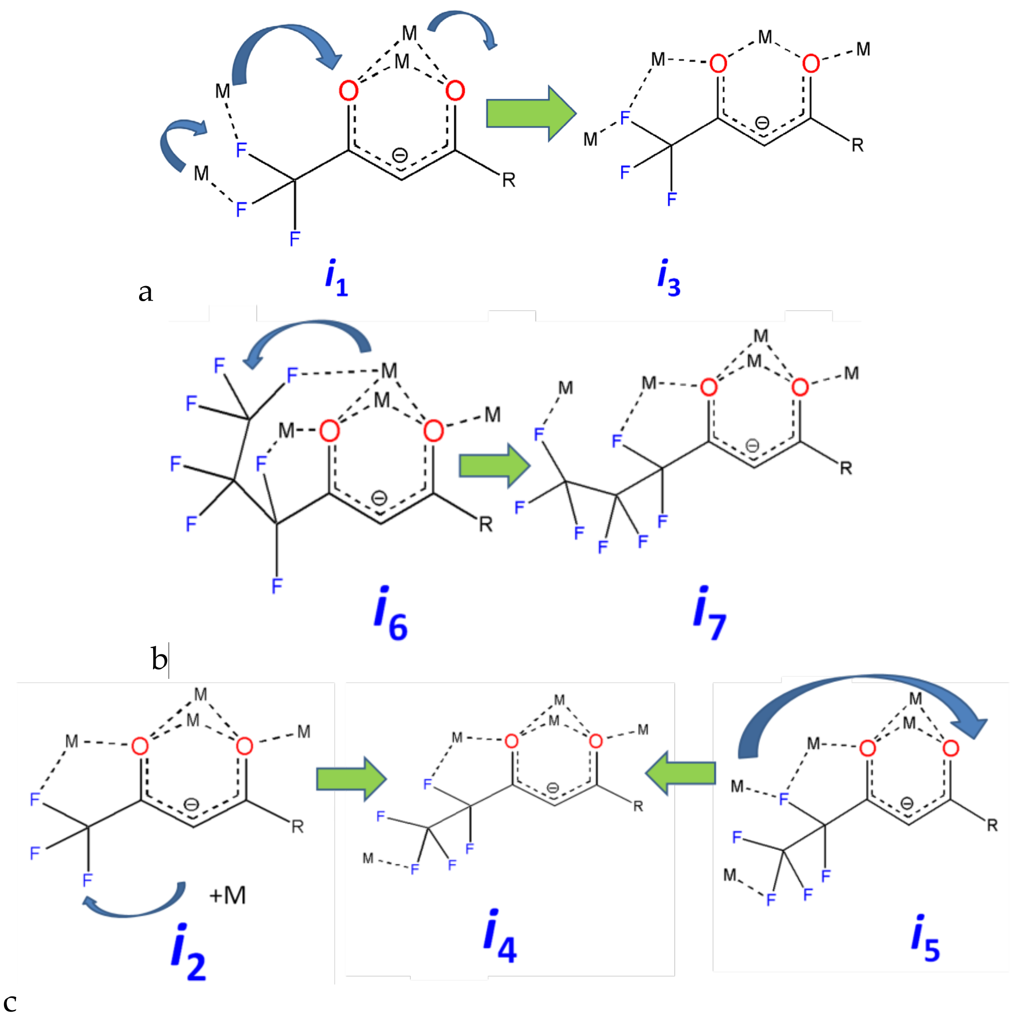

Figure S3. Relationships between the coordination modes of β-diketonate ligands.

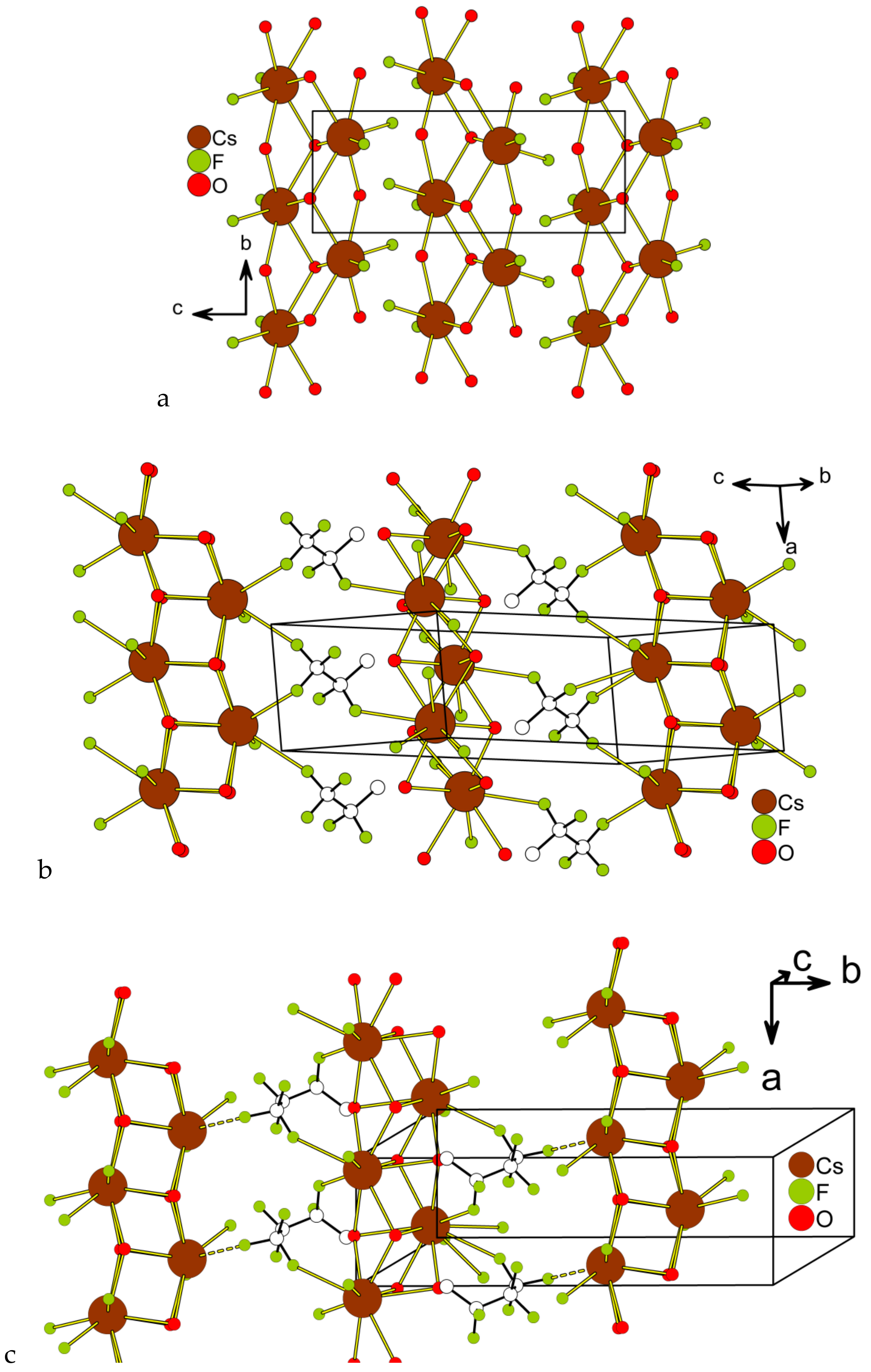

Figure S4. The fragments of the structures of *Cs(Me..CF$_3$)* (a), *Cs(Me..C$_2$F$_5$)* (b) and *Cs(Me..C$_3$F$_7$)* (c). Carbon and hydrogen atoms are omitted.

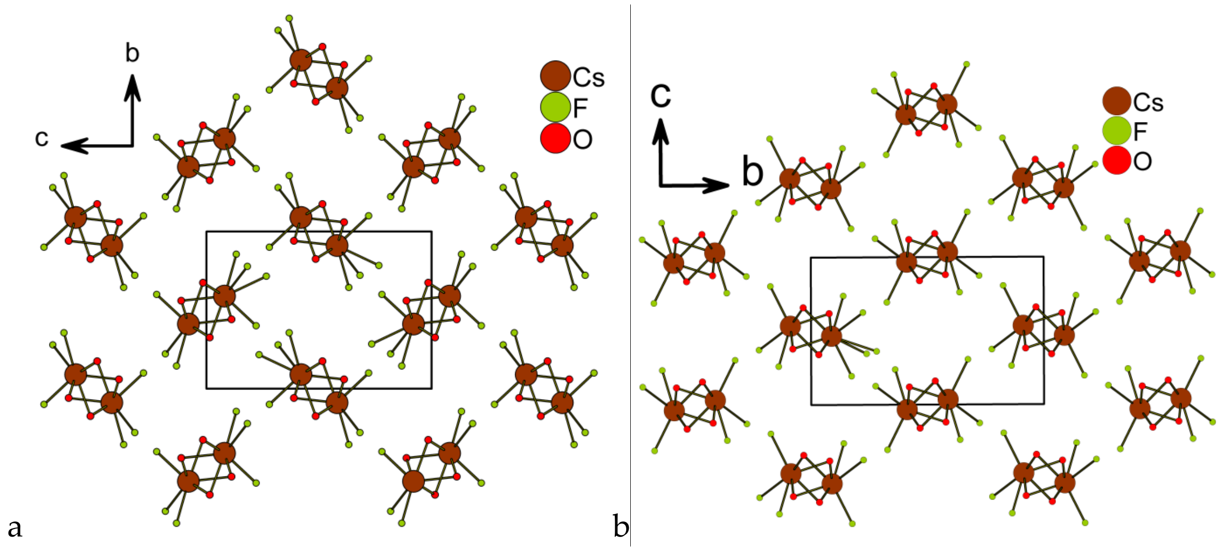

Figure S5. Hexagonal motif of cesium cation packing in the structures *Cs(Me..C*$_2$*F*$_5$*)* (a) and *Cs(Me..C*$_3$*F*$_7$*)* (b).

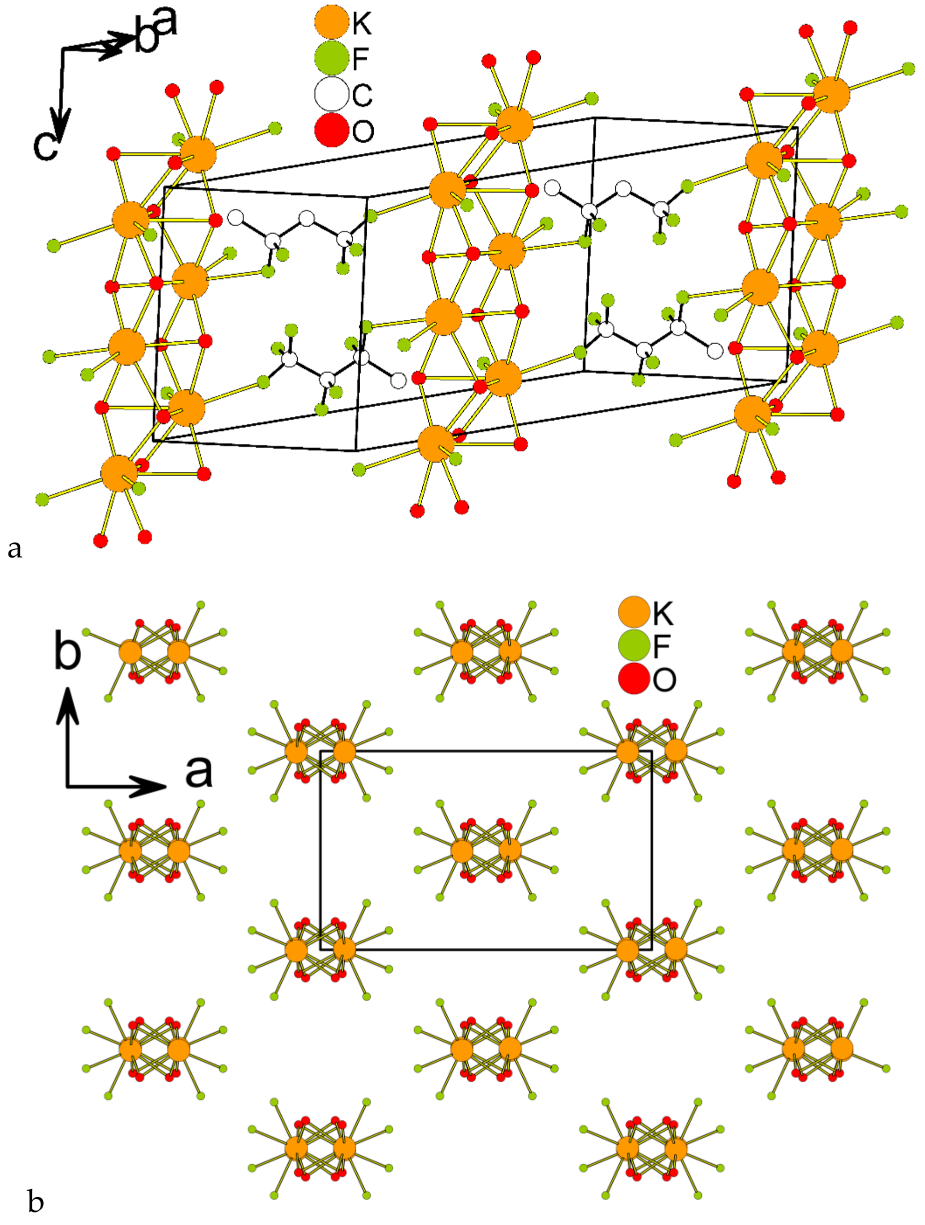

Figure S6. The fragment of "double chains" (a) and hexagonal motif of potassium cation packing (b) in the structure of *K(Me..C*$_3$*F*$_7$*)*.

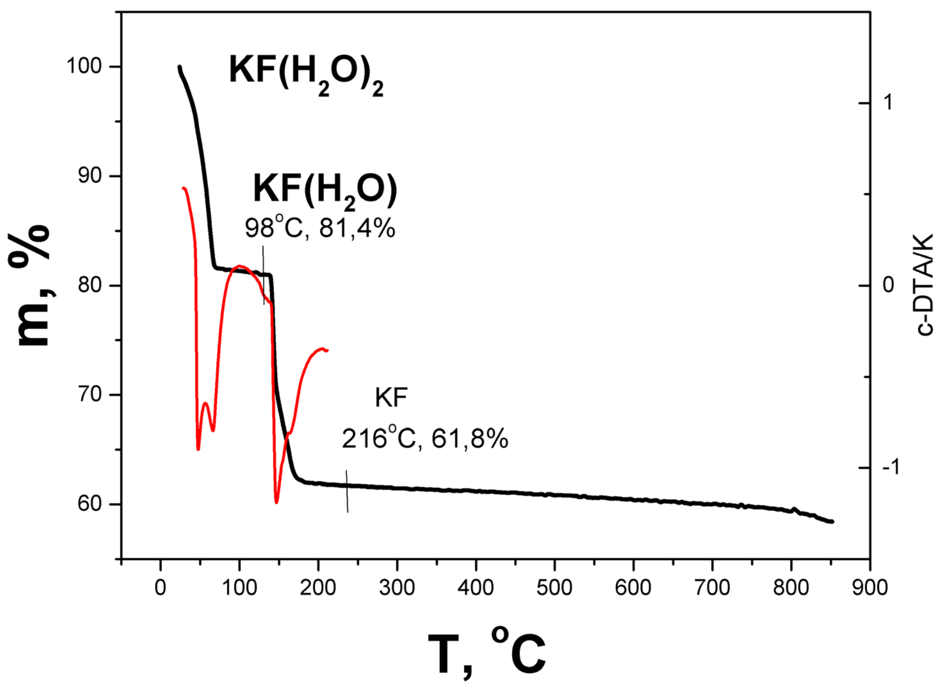

Figure S7. Thermal analysis data for $KF(H_2O)_2$.

Table S1. Crystallographic data and experimental conditions for complexes from **1** to **6**.

Identification	Cs(Me..C_2F_5) (**1**)	Cs(Me..C_3F_7)(H_2O) (**2**)	Cs(Me..C_3F_7) (**3**)	K(Me..C_2F_5)(H_2O) (**4**)	K(Me..C_2F_5) (**5**)	K(Me..C_3F_7) (**6**)
Empirical formula	$C_6H_4F_5O_2Cs$	$C_7H_6F_7O_3Cs$	$C_7H_4F_7O_2Cs$	$C_6H_6F_5O_3K$	$C_6H_4F_5O_2K$	$C_7H_4F_7O_2K$
Formula weight, g·mol^{-1}	336.00	404.03	386.01	260.21	242.19	292.20
Temperature, K	150(2)	220(2)	220(2)	150(2)	220(2)	150(2)
Crystal system	Monoclinic	Monoclinic	Monoclinic	Orthorhombic	Monoclinic	Monoclinic
Space group	$P2_1/n$	$C2/c$	$P2_1/n$	$F222$	$P2_1/n$	$C2/c$
a, Å	5.0200(2)	24.3346(5)	5.2090(2)	10.4169(3)	12.7927(2)	19.5487(9)
b, Å	11.3276(6)	11.1421(3)	17.7467(6)	15.9907(7)	9.9460(2)	11.6001(5)
c, Å	16.2747(8)	18.1783(4)	11.4557(5)	22.8244(11)	15.2075(3)	9.0170(3)
α,°	90	90	90	90	90	90
β, °	95.7280(10)	97.9520(10)	100.5900(10)	90	113.8260(10)	99.1330(10)
γ, °	90	90	90	90	90	90
Volume, Å^3	920.83(8)	4881.4(2)	1040.96(7)	3801.9(3)	1770.04(6)	2018.83(14)
Z	4	16	4	16	8	8
Density (calculated), g/cm^3	2.424	2.199	2.463	1.818	1.818	1.923
Absorption coefficient, mm^{-1}	4.072	3.121	3.646	0.622	0.653	0.618
F(000)	624	3040	720	2080	960	1152
Crystal size, mm^3	0.45 x 0.05 x 0.02	0.21 x 0.20 x 0.16	0.15 x 0.04 x 0.03	0.32 x 0.22 x 0.10	0.21 x 0.19 x 0.08	0.36 x 0.09 x 0.05
Theta range for data collection, °	2.194 to 27.567	2.252 to 27.094	2.142 to 27.512	2.498 to 27.108	2.517 to 27.076	2.048 to 27.557
Index ranges	$-5 \le h \le 6$<br>$-12 \le k \le 14$<br>$-21 \le l \le 20$	$-31 \le h \le 28$<br>$-14 \le k \le 14$<br>$-20 \le l \le 23$	$-6 \le h \le 6$<br>$-23 \le k \le 23$<br>$-14 \le l \le 14$	$-8 \le h \le 12$<br>$-20 \le k \le 20$<br>$-24 \le l \le 29$	$-16 \le h \le 15$<br>$-11 \le k \le 12$<br>$-19 \le l \le 19$	$-25 \le h \le 25$<br>$-15 \le k \le 15$<br>$-11 \le l \le 7$
Reflections collected	6564	19502	11753	4652	16039	7562
Independent reflections	2125	5337	2388	2013	3895	2307
R(int)	0.0312	0.0370	0.0268	0.0193	0.0360	0.0311
Completeness to theta = 25.25°, %	100.0 %	99.3 %	99.6 %	97.6 %	99.9 %	99.7 %
Data / restraints / parameters	2125 / 0 / 129	5337 / 47 / 370	2388 / 0 / 155	2013 / 2 / 148	3895 / 0 / 255	2307 / 0 / 155
Goodness-of-fit on F^2	1.085	1.064	1.228	1.079	1.047	1.065
Final R indices [I > 2sigma(I)]	R_1 = 0.0204<br>wR_2 = 0.0505	R_1 = 0.0335<br>wR_2 = 0.0892	R_1 = 0.0306<br>wR_2 = 0.0683	R1 = 0.0227<br>wR2 = 0.0595	R_1 = 0.0323<br>wR_2 = 0.0769	R_1 = 0.0332<br>wR_2 = 0.0871
R indices (all data)	R_1 = 0.0244<br>wR_2 = 0.0518	R_1 = 0.0460<br>wR_2 = 0.0946	R_1 = 0.0331<br>wR_2 = 0.0691	R_1 = 0.0235<br>wR_2 = 0.0599	R_1 = 0.0403<br>wR_2 = 0.0798	R_1 = 0.0401<br>wR_2 = 0.0909
Largest diff. peak and hole, e·Å^{-3}	0.892 and -0.840	0.953 and -0.779	1.479 and -0.781	0.295 and -0.264	0.318 and -0.253	0.698 and -0.547
Absolute structure parameter				0.06(4)		
CCDC number	2282726	2282727	2282728	2282729	2282730	2282731

Table S2. Polyhedra analysis for potassium and cesium β-diketonate. The lowest residual factors **CShM** are highlighted in grey.

	OP-8	HPY-8	HBPY-8	CU-8	SAPR-8	TDD-8	JGBF-8	JETBPY-8	JBTPR-8	BTPR-8	JSD-8	TT-8	ETBPY-8
**Cs(Me..C_2F_5)**													
Cs1	CCU-9												
**Cs(Me..C_3F_7)(H_2O)**													
Cs1	30.452	23.922	12.019	4.058	8.613	6.577	18.105	27.497	10.443	10.357	11.349	4.694	22.039
Cs2	26.511	16.482	9.183	6.051	7.570	6.484	15.971	21.776	9.714	9.249	10.798	6.769	18.564
Cs3	36.239	23.553	11.594	5.017	2.834	2.939	13.544	25.929	4.288	3.985	5.970	5.862	23.542
**Cs(Me..C_3F_7)**													
Cs1	26.354	8.938	17.980	18.222	13.108	13.338	19.897	25.444	13.842	12.621	15.672	18.372	22.164
**K(Me..C_2F_5)(H_2O)**													
K1	33.185	23.328	12.201	4.182	5.535	3.743	18.727	27.918	7.885	7.249	9.518	4.546	23.401
K2	28.028	20.255	13.651	8.949	3.933	1.819	15.918	27.044	3.759	3.489	4.829	9.786	23.293
K3	32.551	24.273	10.293	3.492	2.700	2.751	15.511	26.255	5.293	4.374	7.172	4.409	23.504
**K(Me..C_2F_5)**													
K1	28.508	18.077	12.639	10.656	5.323	3.461	13.474	26.778	4.833	4.479	6.657	11.330	21.823
K2	CTPR-7												
**K(Me..C_3F_7)**													
K1	30.052	23.130	7.020	3.465	8.549	6.318	13.393	24.708	9.987	8.128	11.365	3.964	21.959
Literature data													
**Cs(Ph..CF_3)(H_2O)**													
Cs1	28.557	21.720	13.065	5.610	6.303	5.135	18.220	28.689	8.063	7.722	9.503	6.388	22.891
Cs2	30.803	25.099	14.017	12.456	3.584	2.069	10.376	27.674	3.166	3.146	2.245	13.278	25.370
**Cs(^{t}Bu..CF_3)(H_2O)**													
Cs1	24.995	20.759	8.147	4.960	7.253	6.501	14.412	22.692	9.261	8.022	10.431	5.657	20.489
Cs2	25.572	21.356	8.654	4.486	7.170	6.583	15.409	21.965	9.383	8.323	10.676	5.199	19.913
**Cs(Me..CF_3)**													
Cs1	27.819	18.723	7.533	7.570	13.126	10.813	10.640	21.300	14.550	13.362	16.193	7.412	18.209
**K(Ph..CF_3)(H_2O)**													
K1	29.845	22.933	12.200	4.473	5.524	3.828	17.369	27.999	6.836	6.833	7.428	4.840	22.529
K2	30.089	22.976	13.013	8.879	2.540	2.310	11.671	23.979	2.618	2.723	2.505	9.741	23.182
**K(Cl-C_6H_4..CF_3)(H_2O)**													
K1	26.378	19.096	7.898	4.356	6.445	5.320	14.748	22.394	8.811	8.186	9.909	5.021	20.160
K2	27.217	18.828	8.139	4.267	6.460	4.924	15.253	22.005	8.616	7.989	9.484	4.911	20.035

Reference shape: OP-8 – Octagon (D_{8h}), HPY-8 – Heptagonal pyramid (C_{7v}), HBPY-8 – Hexagonal bipyramid (D_{6h}), CU-8 – Cube (O_h), SAPR-8 – Square antiprism (D_{4d}), TDD-8 – Triangular dodecahedron (D_{2d}), JGBF-8 – Johnson gyrobifastigium J26 (D_{2d}), JETBPY-8 – Johnson elongated triangular bipyramid J14 (D_{3h}), JBTPR-8 – Biaugmented trigonal prism J50 (C_{2v}), BTPR-8 – Biaugmented trigonal prism (C_{2v}), JSD-8 – Snub diphenoid J84 (D_{2d}), TT-8 – Triakis tetrahedron (T_d), ETBPY-8 – Elongated trigonal bipyramid (D_{3h}). CTPR-7 – Capped trigonal prism (C_{2v}) and CCU-9 – Spherical-relaxed capped cube (C_{4v}).
